# Cases in Which the Moxa Was Successfully Applied

**Published:** 1828-06

**Authors:** R. Wade

**Affiliations:** Member of the Royal College of Surgeons.


THE LONDON
Medical and Physical Journal.

Nº 352, VOL. LIX.] JUNE, 1828. [Nº 24, *New Series*.

For many fortunate discoveries in medicine, and for the detection of numerous errors, the world is indebted to the rapid circulation of Monthly Journals; and there never existed any work, to which the Faculty, in Europe and America, were under deeper obligations, than to the Medical and Physical Journal of London, now forming a long, but an invaluable, series.—RUSH.

### ORIGINAL PAPERS,

AND

### CASES OBTAINED FROM PUBLIC INSTITUTIONS AND OTHER AUTHENTIC SOURCES.

### MOXA.

*Cases in which the Moxa was successfully applied.* By R. WADE, Member of the Royal College of Surgeons.

*Case of Epilepsy.*—ELIZABETH M'DOUGAL, aged seventeen, of delicate appearance, had an epileptic fit (for the first time) about four years ago, in consequence, as she supposes, of a sudden fright; soon after which she complained of pain and giddiness in her head. These symptoms continued increasing, and at the end of a fortnight an epileptic fit came on, which for six months recurred regularly twice or thrice in the twenty-four hours, each attack lasting from an hour to an hour and a half. The paroxysms of the disease became gradually less frequent, and entirely subsided in fifteen months from their commencement. For twelve months she had no return of epilepsy, but during that time suffered much from sickness and frequent palpitations, as well as from occasional pains in the head. Shortly after this period, however, the fits returned, their recurrence being attributed by the patient to her having again been suddenly alarmed. From this time her left side grew weaker, and in three months became entirely paralytic.

I first saw this case in January 1827, and the following notes were taken on the 12th of that month: Pulse 112, small and wiry; extremities very cold; constant pain in the left side, occasionally extending to the back; frequent palpitations; a sense of weight in the head, with giddiness and dimness of sight; a coldness experienced in the right half of the brain, this part feeling heavier than the opposite side; the stomach extremely irritable, rejecting the greater portion of food soon after it has been taken; the bowels constipated, and the left side of the body paralytic.

The Nitrate of Silver had been taken in large doses before I saw the patient, as had also the most powerful tonics and antispasmodics. No good had resulted from the application of leeches and blisters. I was informed that the only relief obtained during the last attack had been from bleeding in the arm; but having taken eight ounces of blood (as much as the patient's strength would admit of), and observing that, although by this means the head and side were slightly relieved, the convulsions became more frequent, the pain also soon returning as severe as before the bleeding, a perseverance in the use of this remedy appeared to be unjustifiable.

An attentive consideration of the symptoms led me to ascribe them principally to a want of force in the arteries of the brain, especially those of the right half of this organ; that the blood, consequently, passing but slowly from the arteries to the veins, a degree of stagnation in the circulation would be the result, readily accounting for the cerebral disturbance, irritability of stomach, and other symptoms. It was this view of the disease which induced me to have recourse to the Moxa, thinking that the application of this powerful agent to the arteries supplying the brain might probably stimulate them to a more healthy action than they at present possessed; for those only who have used this remedy will believe the extent of its influence when the heat is applied in a gradual manner.

The Moxa was first used on the 17th of March, and before its application the following symptoms were noted: Pulse 120, small and weak; extremities cold; lancinating pain extending from the scrobiculus cordis to the back; pain with a sense of weight in the head, and coldness in the right half of the brain; dimness of sight; tongue moist and clean; bowels constipated; extreme irritability of stomach; very little sleep is obtained, and that disturbed by frightful dreams; convulsions generally occur from two to three o'clock in the afternoon, and last for an hour or two, giddiness and dimness of sight being usually their precursors; occasional rigors, and frequent palpitations; the action of the heart is very indistinct; the left side of the body paralytic. The patient often imagines the presence of apparitions, at the same time being conscious of her own delusion. Three moxas were used, one over the junction of the occiput with the atlas, and one to each side of the neck: they were allowed to burn slowly, and held between two and three inches from the part, so as only to produce a slight redness of the skin. The relief obtained was immediate.

March 18th.—The convulsions, instead of occurring in the afternoon, as before the application of the moxa, were deferred until three o'clock the following morning, and continued only half the usual period. Moxas again used as yesterday.

19th.—A fit at four o'clock in the morning. Two moxas applied.

20th.—Fit at half-past two o'clock in the morning, which lasted a quarter of an hour; the pain in the head has almost subsided. Two moxas used.

21st.—No fit; a good night's rest; less sickness. Moxas repeated.

22d.—A fit this morning, which lasted a quarter of an hour. Moxas continued.

23d.—No fit; the sickness much abated, and all the head symptoms subsiding. Moxas as before.

24th.—No fit; pulse eighty-eight, increased in strength.

25th.—A slight attack of the convulsions at one o'clock this morning, attributed by the patient to her having been suddenly awoke. Moxas used.

26th.—No fit. Moxas applied.

27th.—No fit. Moxa continued.

29th.—No fit since the 25th. Moxas applied every day.

30th.—A fit this morning, of a quarter of an hour's duration. Moxas used.

31st.—No fit. Moxas repeated.

April 1st.—No fit. Moxas applied.

2d.—A fit this morning, which continued for ten minutes.

4th.—No fit yesterday or today. Moxas used.

6th.—A fit this morning. Moxas repeated.

10th.—No fit since the 6th; no vomiting for the last two days; the side feels stronger. Moxas used every day.

12th.—No fit: moxas repeated. When these were first used, the sensation of heat continued only for the time of their application: it now, however, remains for a quarter of an hour afterwards.

16th.—No fit; side much stronger. Moxas applied every day.

18th.—A fit at seven o'clock this morning, of ten minutes' duration. Moxas repeated.

21st.—No fit: moxas every day. The irritability of stomach is now very slight.

23d.—The patient had yesterday been much frightened. A fit this morning, which lasted for a few minutes. Moxas applied.

26th.—A fit this morning, which continued five minutes. Moxas used every day.

30th.—A fit this morning, of five minutes' duration: moxas every day. Has been able to walk about her room for the last fortnight; her strength is increasing every day.

May 1st.—No fit. Walked about a quarter of a mile yesterday, after having been unable to leave the house during fifty-one weeks. Moxas applied every day.

7th.—No fit; strength increased. Moxas used every day.

15th.—No fit: moxas continued every day. Has complained, for the last three or four days, of pain in the regio cordis. She was consequently cupped, eight ounces of blood being taken.

16th.—Pain has nearly subsided. Moxas repeated.

20th.—A slight convulsive attack this morning. Moxas every day.

26th.—A fit yesterday, in consequence of extreme mental agitation; occasional pain in the head during the last three days. Moxas repeated every day.

31st.—No fit, or pain in the head; the patient walked yesterday for an hour; the irritability of stomach has entirely ceased. Moxas every day.

June 5th.—No fit: moxas omitted yesterday. Slight pain in the head and giddiness today: moxas repeated; they afforded immediate relief.

August 2d.—No fit; the general health much improved. The moxas have been used latterly only every third day. The patient being desirous of change of air, left town this day.

September 17th.—Has had no return of her fits whilst in the country. Her appearance is much improved. She complains, however, of occasional pain in the head and side, also palpitations. Moxas applied. V.S. ad f℥viij.

October 9th.—No fit: moxas used every third day. Pain in the side and palpitations occasionally come on, but rarely last more than a few hours.

November 12th.—No fit: moxas used every third or fourth day.

19th.—No fit; pain in the side during the last week. V.S. ad f℥viij. Moxas applied as before.

25th.—Much alarmed yesterday by a person coming unexpectedly to see her. Moxas used every third day.

December 27th.—No fit since the last report; no pain in the head or side. Moxas used every third or fourth day.

January 20th, 1828.—No fit: moxas occasionally applied.

February 9th.—No fit; health much improved. Moxas used occasionally.

March 1st.—No fit: one moxa applied every fourth or fifth day.

I saw this patient on the 25th of April. Her health was better than it had been for a very long time: the moxa is, however, still continued once or twice a week.

At the time when the affection of the head was most distressing, the immediate relief afforded by the moxa was extremely satisfactory: the patient says, that after its application her ideas became vivid, and she experienced a throbbing in the right half of the head; a gradual warmth succeeded the coldness, and in a few minutes this side felt like the other.

*Case of Paralysis.*—Elizabeth Cox, aged fifty-seven, was attacked with paralysis of her right side in August 1826. I first saw her early in the following February, and learnt from the patient that she had been subject to pain in the head, and occasional giddiness, for some months previous to the occurrence of the paralytic affection; the power of speech, at first lost, was gradually restored in a few days. The muscles on the right side were quite useless, and scarcely any indications of returning sensation were observed, although the most powerful remedies usually recommended in such cases had been tried. It was this circumstance that induced me, after having persevered, without the slightest degree of success, in the use of stimulating embrocations, blisters, &c., to have recourse to the moxa. Having used this application to the nape of the neck in similar cases with no apparent advantage, I was induced in this instance to apply it to the muscles generally, conceiving that their loss of power might probably at the present time depend more on their own unhealthy state than that of the brain.

March 14th.—A moxa was burnt slowly over the deltoid, and another over the biceps; a slight redness of the skin being produced by their combustion.

15th.—More sensation in the arm: the patient can lift it three or four inches from her sides, although yesterday she had not the slightest power over the muscles. Four moxas applied today, one to the deltoid, and three to the glutei.

16th.—The patient can lift her foot three inches from the ground; she has more sensation in the arm. One moxa to the muscles of the back and loins, and two to the abdominal and glutei muscles.

17th.—Three moxas applied to the same muscles as yesterday. The woman was desired to attempt to walk, with the assistance of her husband: when moving the diseased side, which was effected with a dragging motion, she describes the loss of power as appearing to be chiefly in the lower part of the back and bend of the knee. The foot can now be put flat on the ground.

18th.—Two moxas to the muscles of the back, and one over the rectus femoris. On an attempt being afterwards made to walk, it was evident that the affected side required much less support than yesterday. The good effect of the moxa to the rectus was strikingly observed; for, when the patient was leaning on the right side, there was very little bending of the knee.

19th.—Two moxas to the thigh and leg.

20th.—A moxa to the vastus externus, another to the vastus internus, and another to the patella. The patient has improved much during the last two days: there is now very little bending of the knee, and the attempts to walk are much more successful than before.

21st.—A moxa to the neck, another to the loins, and one to the extensors of the arm.

22d.—A moxa to the knee, and one to the extensors of the forearm.

23d.—Three moxas applied today, one to the deltoid, and two to the extensors and flexors of the thigh. The patient has gained strength during the last four days; she can now raise her hand to her mouth.

25th.—Two moxas applied to the thigh, and one to the arm. Patient walks better.

April 6th.—The moxas have been used every other day. The woman being now able to walk with the assistance of crutches, the moxas were discontinued. She was desired to use the flesh-brush freely, and also to exercise the muscles as much as could be borne without fatigue. From this period her strength gradually increased, and she at present walks very well, having laid aside her crutches some months: there is not, however, so free an action of the muscles on the right as on the left side.

As the description of the case of epilepsy may be considered by many readers more minute than requisite, I beg to observe that my intention, in offering these cases to the notice of the profession, being merely to induce those gentlemen who have similar cases under treatment to give the moxa a fair trial, and not to recommend it as a specific in the cure of epilepsy, (which, in a disease dependent on so many causes, would be absurd,) it appeared far better to incur the charge of tediousness, than, by imperfectly describing the symptoms, to bring the remedy into disrepute, by leading others to employ it in instances perhaps very different to the one in which its powers proved so beneficial. I have only to observe, in conclusion, that the moxa was used in the modified manner recommended by Mr. Boyle in affections of the joints.

*68, Dean-street, Soho;*
*May 8th,* 1828.

